# Supplementary material for: Space-time optical diffraction from synthetic motion
Source: Nat Commun. 2025 Jun 3;16:5147. doi: 10.1038/s41467-025-60159-9 (PMC12134302; doi:10.1038/s41467-025-60159-9)
Supplement: Supplementary file 1 — Supplementary Information [file 41467_2025_60159_MOESM1_ESM.pdf]

Supplementary Information

Space-Time Optical Diffraction from Synthetic Motion

A. C. Harwood, S. Vezzoli, T. V. Raziman, R. Tirole, F. Wu, J. B. Pendry, and R. Sapienza

Blackett Laboratory, Imperial College, Prince Consort Rd,

*South Kensington, London SW7 2BW, UK.**

C. Hooper and S. A. R. Horsley

School of Physics and Astronomy, University of Exeter,

Stocker Road, Exeter, EX4 4QL, UK.

Stefan. A. Maier

School of Physics and Astronomy, Monash University, Clayton Victoria 3800, Australia

This PDF file includes:

Materials and Methods

Supplementary Text

Figs. S1 to S10

* a.harwood22@imperial.ac.uk

SUPPLEMENTARY METHODS

The Sample

The sample is a 40 nm film of Indium Tin Oxide (ITO) (from Präzisions Glas & Optik GmbH) with an epsilon-near-zero frequency of 227 THz (1,320 nm), deposited on a substrate of glass and covered by a 100 nm layer of gold. The gold layer enhances the field confinement and amplifies the reflectivity modulation (measured about 230.2 THz /1,300 nm) induced by changes in the complex refractive index. An identical saturable time-varying mirror have been extensively characterised in ref. [1].

The Optical Setup

We utilise an angularly resolved pump-probe setup (Supplementary Figure 1a), which manipulates ultrafast, near-infrared pulses (230.2 THz /1,300 nm, 225 fs full-width at half-maximum) generated by a PHAROS (Light Conversion) solid-state laser coupled with an ORPHEUS (Light Conversion) optical parametric amplifier. The laser light from the optical parametric amplifier is separated into three beam paths, corresponding to the lower intensity probe line (green) and the two higher intensity pump lines (red), via beam splitters. These three paths are incident upon the sample from three separate angles, 65° for the probe beam and 57° and 73° for the two pump beams, referred to throughout this document as 'pump 1' and 'pump 2' respectively. Two delay stages in the paths of pump 2 and the probe are then used to synchronise the arrival times of pulses from the three paths at the sample.

The optical setup was optimised to investigate the complex space-time dynamics of the pump-induced modulation, requiring the probe to encompass the modulation in space and time. However, since we investigate the synthetic motion of the modulation in a single spatial dimension x, we must also ensure that the modulation appears homogeneous to the probe in the second dimension y, in order to maximise diffraction efficiency in x. To achieve these conditions, separate focusing lenses of 300 mm and 150 mm are used for the pump and probe beams respectively, resulting in the pump beam spot size being approximately twice that of the probe beam at the sample in both x and y. Then the probe beam is filtered by a slit aperture, denoted by * in Supplementary Figure 1a,

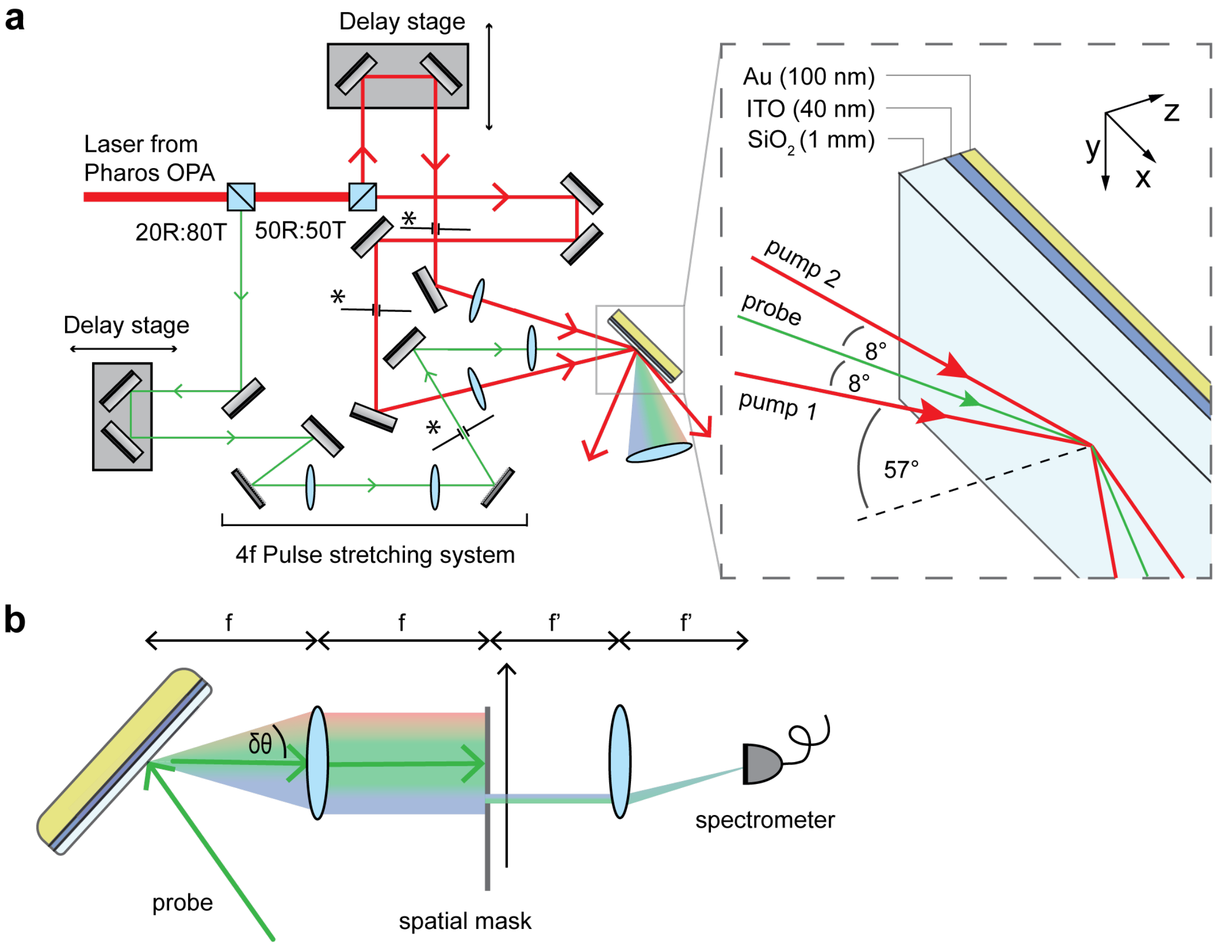

Supplementary Figure 1: The optical setup. (**a**) The pump-probe setup used to generate space-time diffraction from a continuous or discrete forms of synthetic motion via the use of one or two pump beams, which are angularly separable from the far weaker probe beam. (**b**) Space-time diffracted probe light is characterised by a 4f-based detection system that enables the spectral analysis of separate angular components.

placed a focal length from the respective focusing lens to increase the spatial extent of the beam in x, simultaneously decreasing the angular spread of the reflected beam. The use of the slit apertures for manipulating the shape of the pump is also crucial to this experiment and discussed later. In the time domain, we use a 4f pulse-stretching system to increase the duration of the probe beam to approximately three times greater than the pump (see Supplementary Figure 2 for a characterisation), allowing the probe to observe the significant proportion of the modulation in time.

To be able to measure the space-time diffraction of the probe we use a 4f-based detection system, see Supplementary Figure 1b, in order to measure the spectrum of probe light that has been steered to different angles ($\delta\theta$). A spatial mask at the Fourier plane of the first lens (f = 100 mm) is used to angularly select the diffracted light to be then focused by the second lens (f' = 50 mm), before being spectrally analysed using a Princeton Instruments NIRvana infrared spectrometer. To generate our hyperspectral data, the spectrum was measured as a function of a pump-probe delay scan for a range of spatial mask positions across the Fourier plane. Given the focal length of the first lens, a mask of width 0.2 mm enabled an angular resolution of $0.11°$. The displayed plots are taken at the delay with maximal diffraction efficiency, defined as the signal detected outside of the discretised momentum-frequency region within which the reflected probe is detected, divided by the reflected probe signal without pump beam.

Probe Pulse Stretcher

The probe pulses were stretched in time using a 4f pulse stretcher, comprised of two gratings at either side of a symmetric 4f lens setup, see Supplementary Figure 2a. With the use of gratings (G1, G2), the incident pulse may be spatially separated into its spectral components at the Fourier plane of the two lenses (L1, L2) and then recombined. The pulse is stretched in time by an amplitude mask that spectrally filters the pulse transmitted from the Fourier plane. The resultant pulse duration and pulse width, defined as the full–width half maximum of the pulse in time and frequency, was measured for a wide range of mask widths (MW) using cross-correlation between the stretched pulse and the pump pulse in a thin film of Gallium Phosphide, see Supplementary Figure 2b. The mask width used throughout the investigation of continuous synthetic motion and discrete synthetic motion are marked by the blue (MW = 0.25 mm) and orange (MW = 0.6 mm) dashed lines and generated pulses approximately 690 fs and 480 fs in duration. The fitted temporal profile of the pump and probe pulses used, alongside their corresponding spectral profile, are illustrated in Supplementary Figure 2c,d.

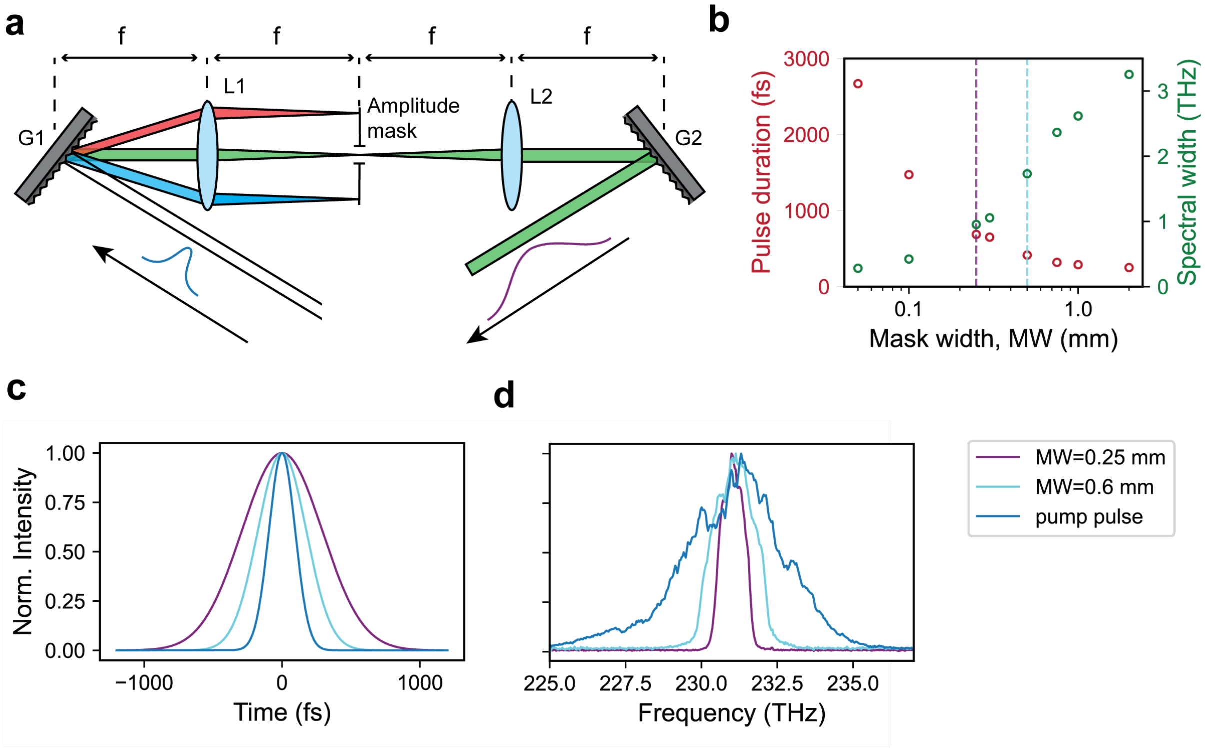

Supplementary Figure 2: The probe pulse stretcher. (**a**) By spatially filtering the Fourier plane of a grating 4f lens system the pulse may be spectrally truncated, resulting in an out going pulse that is longer in time. (**b**) A characterisation of the control enabled over pulse duration and spectral width by tuning the width (MW) of a centralised mask. (**c**) The fitted probe durations and (**d**) spectra used for the studies of continuous synthetic motion (MW = 0.25 mm) and discrete synthetic motion (MW = 0.6 mm), alongside the duration and width of the pump pulse.

Controlling the motion of the Modulation

For a fixed angle, the ratio between the cross-sectional width of the induced modulation in x and its duration in time, defines whether or not the modulation will appear to be in motion. The larger this ratio, the more evident the motion. If the subsequently induced modulation appears to move from the perspective of the traveling probe beam a non-separable space-time diffraction pattern will be measured. When investigating synthetic motion from a single modulation, we leverage this principle and use the slit apertures in the pump beam paths to extend the modulated regions in x, while keeping pulse duration constant. The synthetic motion of the induced modulation is evident from the

non-separable diffraction signature of the probe, which exhibits a characteristic gradient that emerges with increasing pump intensity, see Supplementary Figure 3a. Conversely, to investigate synthetic motion from a pair of modulations, we require individual modulations that do not exhibit motion on their own, but rather exhibit motion when combined. In this case we use only the focused Gaussian beams with a beam width approximately one third of that of the stretched pump beam. The evolution of the subsequently generated diffraction pattern for increasing pump intensity is shown in Supplementary Figure 3b, displaying a much more symmetric, thus separable, diffraction pattern, evidence that there is minimal apparent motion of the modulation.

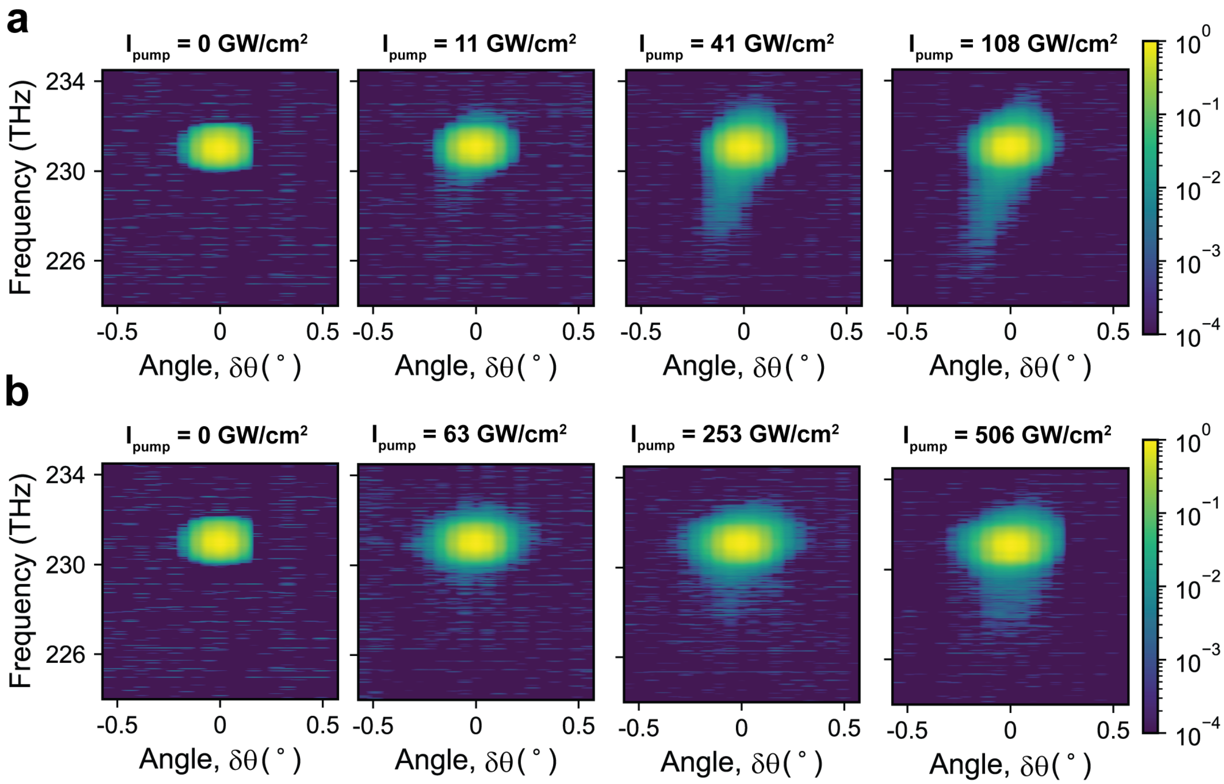

Supplementary Figure 3: Controlling the aspect ratio of the pump beam. (**a-b**) The evolution of the space-time diffraction signature with increasing pump intensity for the case where the pump beam has (**a**) and has not (**b**) been stretched in x. Pump 2 was used in these measurements.

Measuring Modulation Positions

The area and position of the focused spots are measured by diverting beams with a mirror towards a camera placed at the sample plane, perpendicular to the direction of propagation of the probe. The position of the camera along the propagation axis of the probe may be placed with an accuracy of $\pm$1 mm. Due to this uncertainty we calculate a large, 20% uncertainty on the focused pump beam separations for the experiments studying discrete motion. For the separations used in Fig. 3, the pump beams are separated by 520 $\pm$ 104 μm. In the manuscript, we fit the analytical solution (1) to the gradient of the fringes to extract the pump beam separation to be approximately 580 μm, which we later use in our numerical simulations in Figs. 3c, d. Separations of the the pulses in time were reliably set with delay stages. Furthermore, we note that not such fitting is needed for the scheme of continuous motion as these velocities are calculated from the angles of incidence.

Modelling ITO response

Time dependent Drude model

As discussed in [2], a good approximation for the response of strongly pumped ITO to a weaker probe pulse is to use the Drude model with a time–varying plasma frequency. Although Ref. [2] does this within the framework of the Boltzmann equation, here we give a simplified theory that yields the same conclusions. As explained in [3], the origin of the large non-linear response evident in Indium Tin Oxide is the change in the effective mass of the carriers. We thus assume the carriers are subject to Newton's second law, with a time dependent mass, and the force given as a combination of the electric force $q\boldsymbol{E}$ and the collision (damping) force $m(t)\gamma$,

$$\left(\frac{\mathrm{d}}{\mathrm{d}t}[m(t)\boldsymbol{v}] + m(t)\gamma\boldsymbol{v}\right) = q\boldsymbol{E} \tag{1}$$

Here, as discussed in [2], we assume the damping constant γ is time independent. The current density $\boldsymbol{j}$, which is equal to the time derivative of the polarisation is given by the carrier density N, times the electronic charge q, times the velocity $\boldsymbol{v}$,

$$\frac{\partial \boldsymbol{P}}{\partial t} = \boldsymbol{j} = Nq\boldsymbol{v}. \tag{2}$$

We can thus use (1) combined with (2) to derive the differential equation governed by the polarisation of the material,

$$\frac{\partial^2 \boldsymbol{P}}{\partial t^2} = Nq\frac{\mathrm{d}\boldsymbol{v}}{\mathrm{d}t} = -\left(\gamma + \frac{\dot{m}}{m}\right)\frac{\partial \boldsymbol{P}}{\partial t} + \frac{Nq^2}{m(t)}\boldsymbol{E} \tag{3}$$

After application of an integrating factor, the current can be calculated as an integral of the electric field,

$$\begin{aligned}\frac{\partial \boldsymbol{P}}{\partial t} &= \int_{-\infty}^{t} \mathrm{d}t'\,\mathrm{e}^{-\int_{t'}^{t}\left(\gamma+\frac{\dot{m}}{m}\right)\mathrm{d}t''}\frac{Nq^2}{m(t')}\boldsymbol{E}(t') \\ &= \frac{Nq^2}{m(t)}\int_{-\infty}^{t}\mathrm{d}t'\,\mathrm{e}^{-\gamma(t-t')}\boldsymbol{E}(t')\end{aligned} \tag{4}$$

which after a second integration, gives us the polarisation density in the material,

$$\boldsymbol{P}(t) = \epsilon_0 \int_{-\infty}^{t}\mathrm{d}t'\omega_p^2(t')\int_{-\infty}^{t'}\mathrm{d}t''\,\mathrm{e}^{-\gamma(t'-t'')}\boldsymbol{E}(t''), \tag{5}$$

where we have defined the (time dependent) plasma frequency, given by

$$\omega_p^2(t) = \frac{Nq^2}{\epsilon_0 m(t)}. \tag{6}$$

For the theory derived in the next section, we need the time–dependent response of the material to a single frequency of the electric field $\boldsymbol{E}(t) = \boldsymbol{E}_0\mathrm{e}^{-\mathrm{i}\omega t}$. This is given by

$$\begin{aligned}\boldsymbol{P}(t) &= \epsilon_0\int_{-\infty}^{t}\mathrm{d}t'\omega_p^2(t')\int_{-\infty}^{t'}\mathrm{d}t''\,\mathrm{e}^{-\gamma(t'-t'')-\mathrm{i}\omega t''}\boldsymbol{E}_0 \\ &= \epsilon_0\int_{-\infty}^{t}\mathrm{d}t'\frac{\omega_p^2(t')}{\gamma-\mathrm{i}\omega}\mathrm{e}^{-\mathrm{i}\omega t'}\boldsymbol{E}_0\end{aligned} \tag{7}$$

which allows us to identify the permittivity as

$$\epsilon(t,\omega) = \epsilon_\infty + \frac{1}{\gamma-\mathrm{i}\omega}\int_{-\infty}^{t}\mathrm{d}t'\omega_p^2(t')\mathrm{e}^{-\mathrm{i}\omega t'}, \tag{8}$$

where ϵ_∞ represents the 'background' permittivity, which the Drude model tends to at large frequencies. Of course, in reality ϵ tends to unity at very high frequencies, the non–unity value of ϵ_∞ serving to fit the model to experiment, and indicating the presence of higher frequency spectral features that our model does not include. Through integrating by parts, we can expand Eq. (8) as a series, with each successive term depending on a higher order derivative of the plasma frequency

$$\epsilon(t,\omega) = \epsilon_\infty - \frac{1}{\omega(\omega+\mathrm{i}\gamma)}\left[\omega_p^2(t) - \frac{\mathrm{i}}{\omega}\frac{\mathrm{d}\omega_p^2(t)}{\mathrm{d}t} + \left(\frac{\mathrm{i}}{\omega}\right)^2\frac{\mathrm{d}^2\omega_p^2(t)}{\mathrm{d}t^2} - \left(\frac{\mathrm{i}}{\omega}\right)^3\frac{\mathrm{d}^3\omega_p^2}{\mathrm{d}t^3} + \ldots\right]. \tag{9}$$

Thus, taking the relative change in the plasma frequency to be small over a single cycle of the probe (see below)

$$\frac{1}{\omega}\omega_p^{-2}\frac{\mathrm{d}\omega_p^2}{\mathrm{d}t} \ll 1 \tag{10}$$

we can approximate the permittivity as the usual Drude expression, with a time–varying plasma frequency,

$$\epsilon(t,\omega) \sim \epsilon_\infty - \frac{\omega_p^2(t)}{\omega(\omega+\mathrm{i}\gamma)}. \tag{11}$$

We use Eq. (11) in the operator theory given in the next section.

Optical response

We model the significant shift in the permittivity of the ITO layer shown in Supplementary Figure 4a. We write the space–time varying permittivity of the Indium Tin Oxide (ITO) layer in the form $\epsilon_{\mathrm{ITO}}(\boldsymbol{x},t,t-t')$, with the displacement field related to the electric field via the integral relation,

$$\boldsymbol{D}(\boldsymbol{x},t) = \epsilon_0 \int_{-\infty}^{t} \mathrm{d}t' \epsilon_{\mathrm{ITO}}(\boldsymbol{x},t,t-t')\boldsymbol{E}(\boldsymbol{x},t'). \tag{12}$$

Expanding the electric field on the right of Eq. (12) as a Fourier integral, the above expression for the displacement field can be equivalently written as a frequency integral

$$\boldsymbol{D}(\boldsymbol{x},t) = \epsilon_0 \int_{-\infty}^{\infty} \frac{\mathrm{d}\omega}{2\pi} \epsilon_{\mathrm{ITO}}(\boldsymbol{x},t,\omega)\tilde{\boldsymbol{E}}(\boldsymbol{x},\omega)\mathrm{e}^{-\mathrm{i}\omega t}, \tag{13}$$

where the time–frequency dependent permittivity in the above expression is given by,

$$\epsilon_{\mathrm{ITO}}(\boldsymbol{x},t,\omega) = \int_{-\infty}^{t} \mathrm{d}\tau\, \epsilon_{\mathrm{ITO}}(\boldsymbol{x},t,\tau)\mathrm{e}^{\mathrm{i}\omega\tau} = \int_{-\infty}^{\infty} \mathrm{d}\tau\, \epsilon_{\mathrm{ITO}}(\boldsymbol{x},t,\tau)\mathrm{e}^{\mathrm{i}\omega\tau}. \tag{14}$$

For ITO we represent this mixed time–frequency response of ITO using a Drude model, with a constant scattering rate γ but where the plasma frequency ω_p is a function of time (see (11) and preceding text),

$$\epsilon_{\mathrm{ITO}}(\boldsymbol{x},t,\omega) = \epsilon_\infty - \frac{\omega_p^2(\boldsymbol{x},t)}{\omega(\omega+\mathrm{i}\gamma)}. \tag{15}$$

In our calculations we take the background permittivity as $\epsilon_\infty = 3.9$ and the damping rate as $\gamma = 130$ THz (reported values vary in the literature, see Refs [2], [3], and [4]). In the experiment, the time variation of the plasma frequency is due to the interaction of the

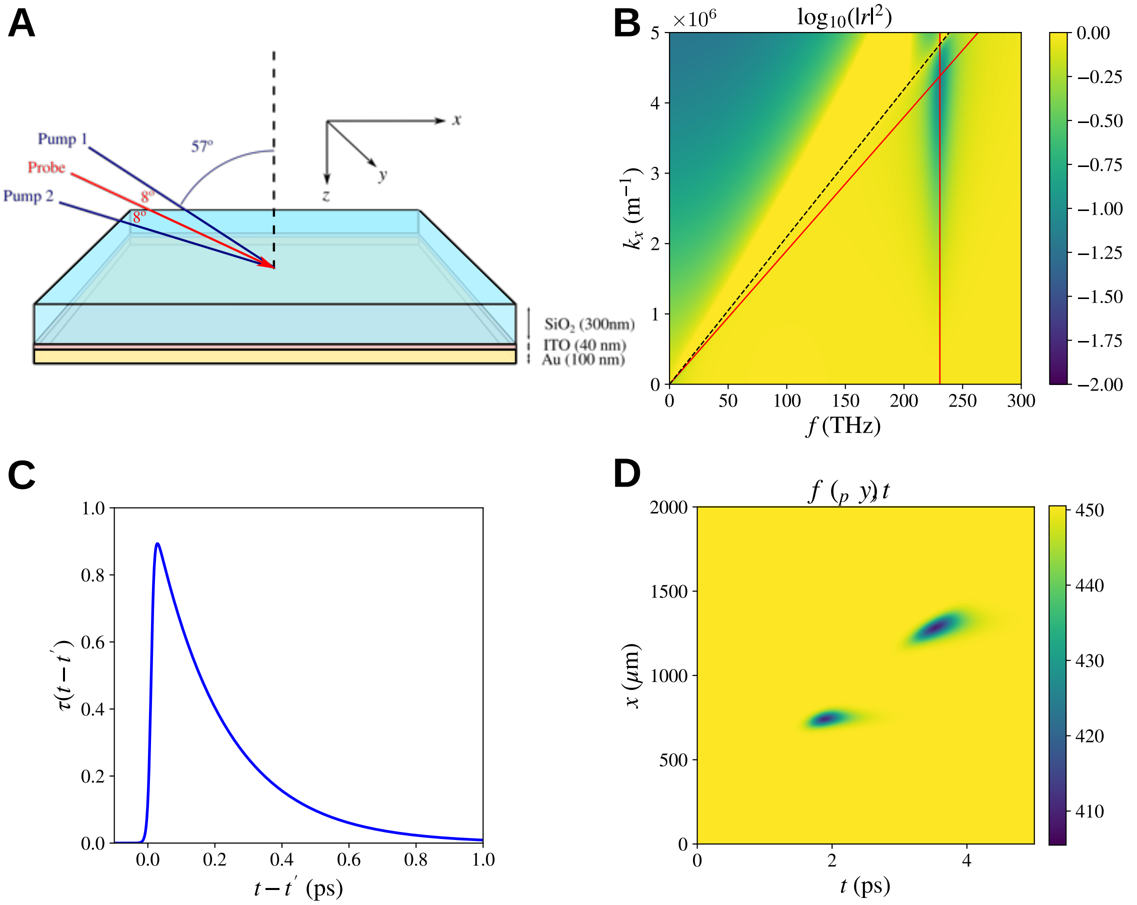

Supplementary Figure 4: **Theoretical model:** (a) we model the sample as three layers; 100 nm Au , 40 nm ITO, and 300 nm SiO_2. The reduced thickness of SiO_2 compared to experiment avoids rapid interference oscillations in Fourier space, a simplification that leads to a small change in the amplitude of the reflected signal. The incident field is set up to consist of two intense pump pulses, pump 1 and pump 2, at $57°$ and $73°$ to the surface normal respectively. The weaker probe pulse is incident at $65°$, where in the zero modulation case the reflectivity of the multilayer is very low, as indicated by the intersection of the two red lines in panel (b) (these lines indicate fixed angle of $65°$ and frequency of $230\,\mathrm{THz}$ within the frequency/wavevector space). (c) The response kernel $\tau(t-t')$ given by Eq. (17), which is convolved with the incident pulse intensity according to Eq. (16) in order to generate the space–time dynamics of the ITO plasma frequency. (d) An example result of this convolution, the modulated plasma frequency $f_p = \omega_p/2\pi$ of the ITO layer entering the operator theory described below.

applied pump field with the electrons in the material. We take the space–time variation of

the plasma frequency to have the phenomenological form

$$\omega_p^2(\boldsymbol{x},t) = \omega_{p,i}^2 + \Delta\omega^2 \sum_{t'} \tau(t-t') I(\boldsymbol{x},t') \tag{16}$$

where $\Delta\omega^2$ is the shift in the square of the plasma frequency (note: *not* the square of the shift), $I(\boldsymbol{x},t)$ is the pump intensity as a function of space and time, the sum is taken over all the time points in the simulation, and the response kernel $\tau(t-t')$ is given by,

$$\tau(t-t') = \frac{1}{2N}\left[1+\tanh\left(\frac{t-t'}{t_{\mathrm{rise}}}-1\right)\right] \mathrm{e}^{-\left(\frac{t-t'}{t_{\mathrm{decay}}}-1\right)}. \tag{17}$$

as shown in Supplementary Figure 4c. In our simulations we use a rise time of $t_{\mathrm{rise}} = 10\mathrm{fs}$ and decay time of $t_{\mathrm{decay}} = 210\mathrm{fs}$. The unmodulated plasma frequency is taken as $\omega_{p,i} = 450.5\,\mathrm{THz}$, and the maximum shift in the plasma frequency is $\Delta\omega_p = -0.1\,\omega_{p,i}$. The time response kernel (17) is of a very similar form to that used in [5] albeit with a shortened rise time ($40\,\mathrm{fs} \to 10\,\mathrm{fs}$) and increased magnitude shift in the plasma frequency ($20\,\mathrm{THz} \to 45\,\mathrm{THz}$), chosen to account for differences in the sample quality. As shown in Supplementary Figure 5, these changes in time and frequency scales do not significantly affect our results. The normalisation constant N in Eq. (17) is chosen so that the maximum value of the summation in Eq. (16) is unity, ensuring that the peak change of the plasma frequency is given by $\omega_{p,\mathrm{peak}} = \omega_{p,i} + \Delta\omega$.

The Au and SiO_2 layers (see Supplementary Figure 4A) have a weak dependence on the pump field and are taken as space and time independent, with permittivities taken from *(36)*

$$\begin{aligned} \epsilon_{\mathrm{SiO_2}}(\omega) &= 1.5 \\ \epsilon_{\mathrm{Au}}(\omega) &= -298.89 + 41.06\mathrm{i} + (1.10\times10^{-13} - 1.58\times10^{-14}\mathrm{i})\,\omega. \end{aligned} \tag{18}$$

Operator theory of space–time diffraction

To calculate reflection from the space–time modulated multilayer shown in Supplementary Figure 4a, with unmodulated reflectivity given in Supplementary Figure 4b, we use an extension of the operator method described in [6]. Here we write the modulated dispersive permittivity of the ITO defined in Eq. (15) as an operator that acts on the frequency

and wavevector electric field distribution $\tilde{E}(k, z, \omega)$,

$$\begin{aligned}
\boldsymbol{D}(x,z,t) &= \epsilon_0 \int_{-\infty}^{\infty} \frac{\mathrm{d}\omega}{2\pi} \epsilon(x,t,\omega) \tilde{\boldsymbol{E}}(x,z,\omega) \mathrm{e}^{-\mathrm{i}\omega t} \\
&= \epsilon_0 \int_{-\infty}^{\infty} \frac{\mathrm{d}\omega}{2\pi} \int_{-\infty}^{\infty} \frac{\mathrm{d}k}{2\pi} \tilde{\boldsymbol{E}}(k,z,\omega) \hat{\epsilon}(-\mathrm{i}\partial_k, \mathrm{i}\partial_\omega, \omega) \mathrm{e}^{\mathrm{i}(kx-\omega t)} \\
&= \epsilon_0 \int_{-\infty}^{\infty} \frac{\mathrm{d}\omega}{2\pi} \int_{-\infty}^{\infty} \frac{\mathrm{d}k}{2\pi} \left[\hat{\epsilon}(\mathrm{i}\partial_k, -\mathrm{i}\partial_\omega, \omega) \tilde{\boldsymbol{E}}(k,z,\omega) \right] \mathrm{e}^{\mathrm{i}(kx-\omega t)},
\end{aligned} \tag{19}$$

where the operator ordering in the permittivity operator is such that all frequency derivatives appear to the *left* of its frequency dependence. Using this notation we can write the Fourier components of the displacement field as

$$\tilde{\boldsymbol{D}}(k,z,\omega) = \epsilon_0 \hat{\epsilon}(\mathrm{i}\partial_k, -\mathrm{i}\partial_\omega, \omega) \tilde{\boldsymbol{E}}(k,z,\omega). \tag{20}$$

Numerically the permittivity operator is constructed using a Fourier transform, i.e. $\hat{\epsilon}(\mathrm{i}\partial_k, -\mathrm{i}\partial_\omega, \omega) = (\mathcal{F}_k \otimes \mathcal{F}_\omega)\, \epsilon(y,t,\omega) \left(\mathcal{F}_\omega^{-1} \otimes \mathcal{F}_k^{-1}\right)$. In terms of this operator notation, the magnetic field can be eliminated from Maxwell's equations, reducing them to the form

$$\boldsymbol{\nabla}\left(\boldsymbol{\nabla} \cdot \tilde{\boldsymbol{E}}\right) - \boldsymbol{\nabla}^2 \tilde{\boldsymbol{E}} - \left(\frac{\omega}{c}\right)^2 \hat{\epsilon} \tilde{\boldsymbol{E}} = 0 \tag{21}$$

In our theory we approximate the permittivity on the surface of the ITO by assuming it has translational invariance along the x axis. This assumption means that we can take the polarisation (transverse magnetic (TM) or transverse electric (TE)) to be conserved. For a TM polarised wave $\boldsymbol{H} = H\boldsymbol{e}_y$, Eq. (21) can be written as a pair of coupled equations for the two components (x and z) of the electric field,

$$\tilde{E}_z = \mathrm{i}\left[\left(\frac{\omega}{c}\right)^2 \hat{\epsilon} - k^2\right]^{-1} k \frac{\mathrm{d}\tilde{E}_x}{\mathrm{d}z} \tag{22}$$

and

$$\mathrm{i}k \frac{\mathrm{d}\tilde{E}_z}{\mathrm{d}z} - \left[\frac{\mathrm{d}^2}{\mathrm{d}z^2} + \left(\frac{\omega}{c}\right)^2 \hat{\epsilon}\right] \tilde{E}_x = 0 \tag{23}$$

Combining the above two equations, (22) and (23), we can derive a single differential equation for the in–plane y component of the electric field,

$$\frac{\mathrm{d}}{\mathrm{d}z}\left(1 + k\left[\left(\frac{\omega}{c}\right)^2 \hat{\epsilon} - k^2\right]^{-1} k\right) \frac{\mathrm{d}\tilde{E}_x}{\mathrm{d}z} + \left(\frac{\omega}{c}\right)^2 \hat{\epsilon} \tilde{E}_x = 0. \tag{24}$$

We split our system into a set of layers homogeneous in z, with the time variation restricted to the ITO, analogous to the well–known transfer matrix technique. In this layer the above equation (24) reduces to

$$\frac{\mathrm{d}^2\tilde{E}_x}{\mathrm{d}z^2}+\left[\left(\frac{\omega}{c}\right)^2\hat{\epsilon}-k\hat{\epsilon}^{-1}k\hat{\epsilon}\right]\tilde{E}_x=0. \tag{25}$$

As described in Ref. [6] we can write the solution to Eq. (25) in terms of the wave–vector operator $\hat{K}$, the square of which is $\hat{K}^2=\left(\frac{\omega}{c}\right)^2\hat{\epsilon}-k\hat{\epsilon}^{-1}k\hat{\epsilon}$. Using this idea, within the space–time varying layer the electric field is given by

$$\tilde{E}_x(k,z,\omega)=\mathrm{e}^{\mathrm{i}\hat{K}z}E^{(+)}(k,\omega)+\mathrm{e}^{-\mathrm{i}\hat{K}z}E^{(-)}(k,\omega) \tag{26}$$

where $E^{(\pm)}$ are the (ω,k) spectra of the right and left–going parts of the field at $z=0$. Applying Maxwell's equations we find that the magnetic field frequency and wavevector spectrum is similarly given by

$$\eta_0\tilde{H}_y(k,z,\omega)=-k_0\hat{\epsilon}\hat{K}^{-1}\left(\mathrm{e}^{\mathrm{i}\hat{K}z}E^{(+)}(k,\omega)-\mathrm{e}^{-\mathrm{i}\hat{K}z}E^{(-)}(k,\omega)\right). \tag{27}$$

Note that, as discussed in [6], in space–time varying systems it is subtle to divide a field into left and right–going parts. This is because time modulated properties can change the sign of the frequency, reversing the direction of propagation while keeping the sign of the wave–vector unchanged. We take the operator square root of $\hat{K}^2$ such that the imaginary part of every eigenvalue of $\hat{K}$ is positive, ensuring all right–going waves decay as they propagate into the medium. Note that this choice of matrix square root does not affect the predictions of the theory, provided we always deal with finite–thickness media. In any case, positive to negative frequency conversion is negligible for the parameters in this experiment.

Single layer reflection and transmission operators

To apply the above operator method to our multilayer (see Supplementary Figure 4a) we first construct the reflection and transmission operators for each of the three layers, assuming each layer is surrounded by vacuum. Using the above expressions, (26) and (27) for the electric and magnetic fields, at the interface between two media (passing from

1 to 2), the continuity of the electric and magnetic fields implies the relations,

$$\begin{aligned} E_1^{(+)} + E_1^{(-)} &= E_2^{(+)} + E_2^{(-)} \\ \hat{\epsilon}_1 \hat{K}_1^{-1} \left(E_1^{(+)} - E_1^{(-)} \right) &= \hat{\epsilon}_2 \hat{K}_2^{-1} \left(E_2^{(+)} - E_2^{(-)} \right). \end{aligned} \tag{28}$$

The above pair of operator equations can be written in matrix form relating the fields in medium 2 in terms of those in medium 1

$$\begin{pmatrix} E_2^{(+)} \\ E_2^{(-)} \end{pmatrix} = \frac{1}{2} \begin{pmatrix} 1+\hat{Z}_{12} & 1-\hat{Z}_{12} \\ 1-\hat{Z}_{12} & 1+\hat{Z}_{12} \end{pmatrix} \begin{pmatrix} E_1^{(+)} \\ E_1^{(-)} \end{pmatrix} \tag{29}$$

where $\hat{Z}_{12} = \hat{K}_2 \hat{\epsilon}_2^{-1} \hat{\epsilon}_1 \hat{K}_1^{-1}$ plays the role of the impedance. Similarly the frequency spectrum of the fields at $z = d$ in medium 2 is related to the spectrum at $z = 0$ in the same medium as follows

$$\begin{pmatrix} E_2^{(+)}(d) \\ E_2^{(-)}(d) \end{pmatrix} = \begin{pmatrix} \mathrm{e}^{\mathrm{i}\hat{K}_2 d} & 0 \\ 0 & \mathrm{e}^{-\mathrm{i}\hat{K}_2 d} \end{pmatrix} \begin{pmatrix} E_2^{(+)}(0) \\ E_2^{(-)}(0) \end{pmatrix}. \tag{30}$$

Combining the above 'boundary crossing' matrices (29) and the propagation matrix (30) we can thus find the relationship for the fields $E_{R,L}^{(\pm)}$ either side of a single layer of material, surrounded by vacuum

$$\begin{aligned} \begin{pmatrix} E_R^{(+)} \\ E_R^{(-)} \end{pmatrix} &= \frac{1}{4} \begin{pmatrix} 1+\hat{Z}_{21} & 1-\hat{Z}_{21} \\ 1-\hat{Z}_{21} & 1+\hat{Z}_{21} \end{pmatrix} \begin{pmatrix} \mathrm{e}^{\mathrm{i}\hat{K}_2 d} & 0 \\ 0 & \mathrm{e}^{-\mathrm{i}\hat{K}_2 d} \end{pmatrix} \begin{pmatrix} 1+\hat{Z}_{12} & 1-\hat{Z}_{12} \\ 1-\hat{Z}_{12} & 1+\hat{Z}_{12} \end{pmatrix} \begin{pmatrix} E_L^{(+)} \\ E_L^{(-)} \end{pmatrix} \\ &= \frac{1}{4} \begin{pmatrix} 1+\hat{Z}_{21} & 1-\hat{Z}_{21} \\ 1-\hat{Z}_{21} & 1+\hat{Z}_{21} \end{pmatrix} \begin{pmatrix} \mathrm{e}^{\mathrm{i}\hat{K}_2 d}(1+\hat{Z}_{12}) & \mathrm{e}^{\mathrm{i}\hat{K}_2 d}(1-\hat{Z}_{12}) \\ \mathrm{e}^{-\mathrm{i}\hat{K}_2 d}(1-\hat{Z}_{12}) & \mathrm{e}^{-\mathrm{i}\hat{K}_2 d}(1+\hat{Z}_{12}) \end{pmatrix} \begin{pmatrix} E_L^{(+)} \\ E_L^{(-)} \end{pmatrix} \\ &= \begin{pmatrix} \hat{\mathcal{T}}_{11} & \hat{\mathcal{T}}_{12} \\ \hat{\mathcal{T}}_{21} & \hat{\mathcal{T}}_{22} \end{pmatrix} \begin{pmatrix} E_L^{(+)} \\ E_L^{(-)} \end{pmatrix}. \end{aligned} \tag{31}$$

where the elements of the operator valued transfer matrix, $\hat{\mathcal{T}}_{ij}$, are given by

$$\begin{aligned} \hat{\mathcal{T}}_{11} &= \frac{1}{4} \left[(1+\hat{Z}_{21})\mathrm{e}^{\mathrm{i}\hat{K}_2 d}(1+\hat{Z}_{12}) + (1-\hat{Z}_{21})\mathrm{e}^{-\mathrm{i}\hat{K}_2 d}(1-\hat{Z}_{12}) \right] \\ \hat{\mathcal{T}}_{12} &= \frac{1}{4} \left[(1+\hat{Z}_{21})\mathrm{e}^{\mathrm{i}\hat{K}_2 d}(1-\hat{Z}_{12}) + (1-\hat{Z}_{21})\mathrm{e}^{-\mathrm{i}\hat{K}_2 d}(1+\hat{Z}_{12}) \right] \\ \hat{\mathcal{T}}_{21} &= \frac{1}{4} \left[(1-\hat{Z}_{21})\mathrm{e}^{\mathrm{i}\hat{K}_2 d}(1+\hat{Z}_{12}) + (1+\hat{Z}_{21})\mathrm{e}^{-\mathrm{i}\hat{K}_2 d}(1-\hat{Z}_{12}) \right] \\ \hat{\mathcal{T}}_{22} &= \frac{1}{4} \left[(1+\hat{Z}_{21})\mathrm{e}^{-\mathrm{i}\hat{K}_2 d}(1+\hat{Z}_{12}) + (1-\hat{Z}_{21})\mathrm{e}^{\mathrm{i}\hat{K}_2 d}(1-\hat{Z}_{12}) \right]. \end{aligned} \tag{32}$$

We find the reflection and transmission amplitudes for waves propagating through this layer through imposing e.g. incidence from the left: $E_R^{(-)} = 0$, $E_R^{(+)} = \hat{T} E_L^{(+)}$, and $E_L^{(-)} =$

$\hat{R}E_L^{(+)}$. Substituting this imposed relation between field amplitudes into (31) leads to the following expressions for the reflection ($\hat{R}$) and transmission ($\hat{T}$) operators,

$$\begin{aligned}\hat{R} &= -\hat{\mathcal{T}}_{22}^{-1}\hat{\mathcal{T}}_{21} \\ \hat{T} &= \hat{\mathcal{T}}_{11} - \hat{\mathcal{T}}_{12}\hat{\mathcal{T}}_{22}^{-1}\hat{\mathcal{T}}_{21}\end{aligned} \tag{33}$$

and similarly for incidence from the right,

$$\begin{aligned}\hat{\bar{R}} &= \hat{\mathcal{T}}_{12}\hat{\mathcal{T}}_{22}^{-1} \\ \hat{\bar{T}} &= \hat{\mathcal{T}}_{22}^{-1}\end{aligned} \tag{34}$$

where we have used the notation of an overbar to indicate incidence from the reverse side. Although the reflection symmetry of a single slab implies the equality for the operators, (33) and (34), we construct the reflection and transmission operators for the multilayer sketched in Supplementary Figure 4a through an iterative process of combining pairs of isolated layers described by (31) and (32) (see next subsection). These combined layers have no reflection symmetry, it thus becomes important to distinguish right and left incidence.

Combining reflection and transmission operators

As described above, we calculate the reflection and transmission operators of the multilayer in terms of the operators of the individual layers, (33) and (34). We assume we have computed the reflection operators $\hat{R}_{1,2}$ and $\hat{\bar{R}}_{1,2}$ for materials, 1 and 2, surrounded by vacuum, for incidence from the left and right respectively, as well as the transmission operators, $\hat{T}_{1,2}$ and $\hat{\bar{T}}_{1,2}$. This could be, for example, the ITO and SiO_2 layers shown in Supplementary Figure 4a. We can construct the reflection and transmission operators for the combination of these two layers (medium 1 placed immediately on the *left* of medium 2) via a geometric series representing the reflections from each interface, the reflection ($\hat{R}$) and transmission ($\hat{T}$) operators for the bi–layer being given by

$$\begin{aligned}\hat{R} &= \hat{R}_1 + \hat{\bar{T}}_1\hat{R}_2\hat{T}_1 + \hat{\bar{T}}_1\hat{R}_2\hat{\bar{R}}_1\hat{R}_2\hat{T}_1 + \cdots = \hat{R}_1 + \hat{\bar{T}}_1\left(1 - \hat{R}_2\hat{\bar{R}}_1\right)^{-1}\hat{R}_2\hat{T}_1 \\ \hat{\bar{R}} &= \hat{\bar{R}}_2 + \hat{T}_2\hat{\bar{R}}_1\hat{\bar{T}}_2 + \hat{\bar{T}}_1\hat{\bar{R}}_1\hat{R}_2\hat{\bar{R}}_1\hat{\bar{T}}_2 + \cdots = \hat{\bar{R}}_2 + \hat{T}_2\left(1 - \hat{\bar{R}}_1\hat{R}_2\right)^{-1}\hat{\bar{R}}_1\hat{\bar{T}}_2\end{aligned} \tag{35}$$

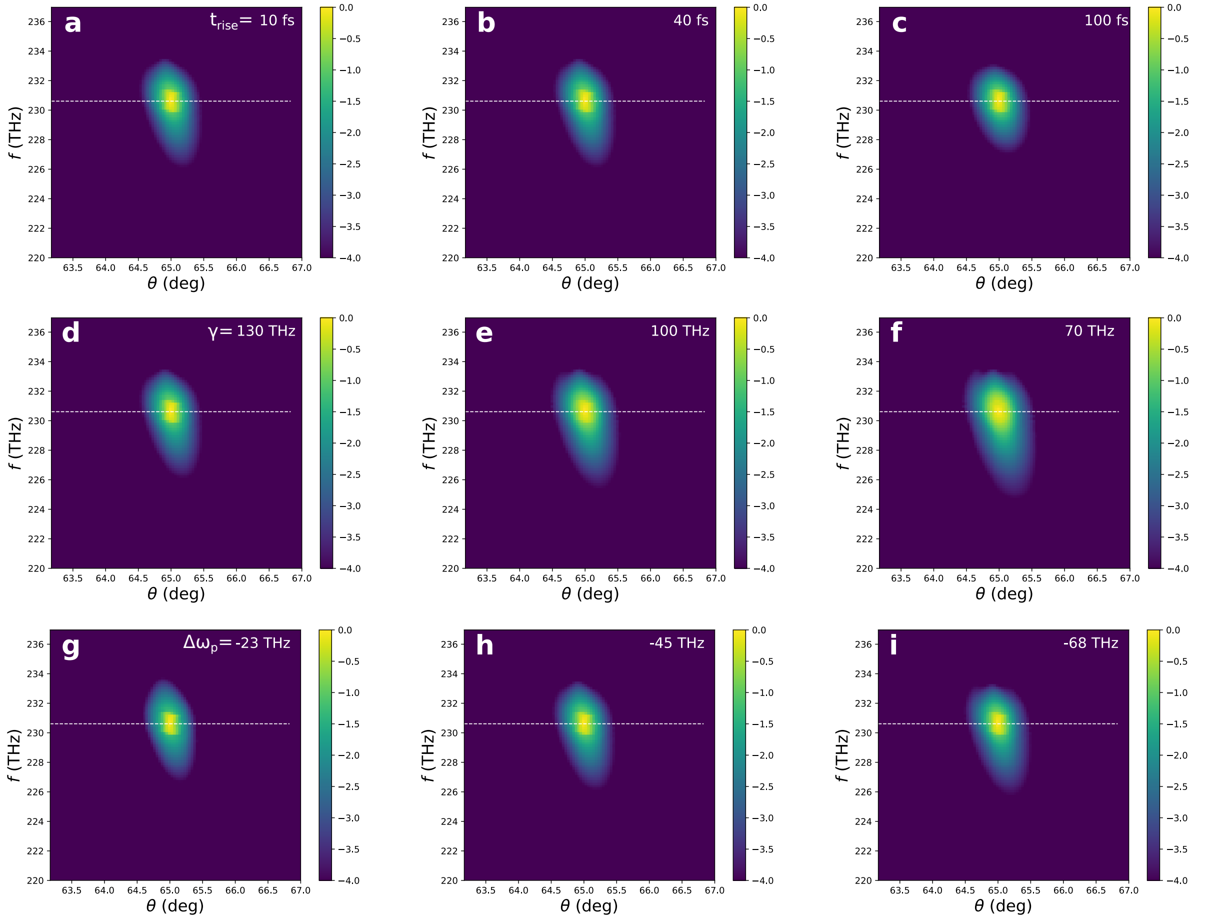

Supplementary Figure 5: **Exploration of frequency and time scales:** Single patch diffraction for a pump incident at $57°$. Panels (**a–c**) show the effect of increasing the rise time of the plasma frequency, t_{rise} given in Eq. (17) showing the very minor effect of the timescale choice discussed above. Panels (**d–f**) show the effect of reducing the damping rate, the value of which is sample dependent [4]. Panels (**f–i**) show the effect of changing the maximum shift in the plasma frequency due to the incident pump pulse. Other parameters are as given in the main text.

and

$$\hat{T} = \hat{T}_2\hat{T}_1 + \hat{T}_2\hat{\hat{R}}_1\hat{R}_2\hat{T}_1 + \hat{T}_2\left(\hat{\hat{R}}_1\hat{R}_2\right)^2\hat{T}_1 + \cdots = \hat{T}_2\left(1-\hat{\hat{R}}_1\hat{R}_2\right)^{-1}\hat{T}_1$$
$$\hat{\hat{T}} = \hat{\hat{T}}_1\hat{\hat{T}}_2 + \hat{\hat{T}}_2\hat{R}_2\hat{\hat{R}}_1\hat{\hat{T}}_2 + \hat{T}_2\left(\hat{R}_2\hat{\hat{R}}_1\right)^2\hat{\hat{T}}_2 + \cdots = \hat{T}_2\left(1-\hat{R}_2\hat{\hat{R}}_1\right)^{-1}\hat{T}_1. \qquad (36)$$

Equations (35) and (36) can be used to build up the reflection and transmission operators for any multilayer, combining the operators (31) in nested pairs. Note that this iterative method is stable, although slower than multiplication of component transfer matrices *(37)*.

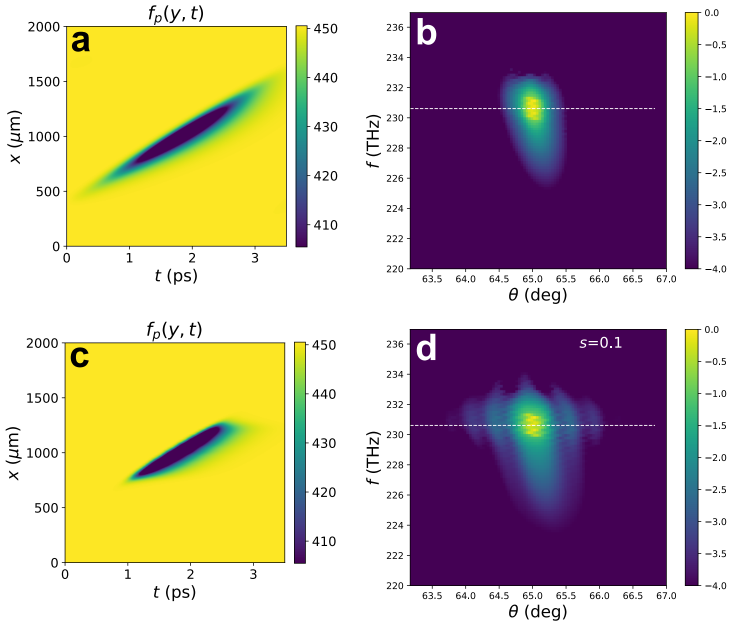

Supplementary Figure 6: **Increasing spread of frequency–momentum diffraction with short time-scale plasma frequency dynamics:** (a–b) Plasma frequency and diffraction pattern for $57°$ pump using theory and parameters described in the text above ($t_{\text{rise}} = 1\text{fs}$). (c–d) as in panels a–b, but for a pump profile that has been further steepened through passing the profile f through the function $\frac{1}{2}\left(1 + \tanh((f - 0.5)/s)\right)$ with $s = 0.1$. In both cases the diffraction pattern extends further in frequency compared to the results given in the main text.

To calculate the diffraction from our system we apply the above method to construct the reflection and transmission operators for the space-time dependent properties of the Au/ITO/SiO_2 multilayer. We then applied the resulting operators to the ω,k spectrum of the probe beam, converting to frequency and angle θ using $k = (\omega/c)\sin(\theta)$.

Approximate theory of space–time diffraction

To gain a simple understanding of the experimental results and the above, somewhat involved numerical solution, we here construct a simple model to explain the main features of the space–time diffraction pattern.

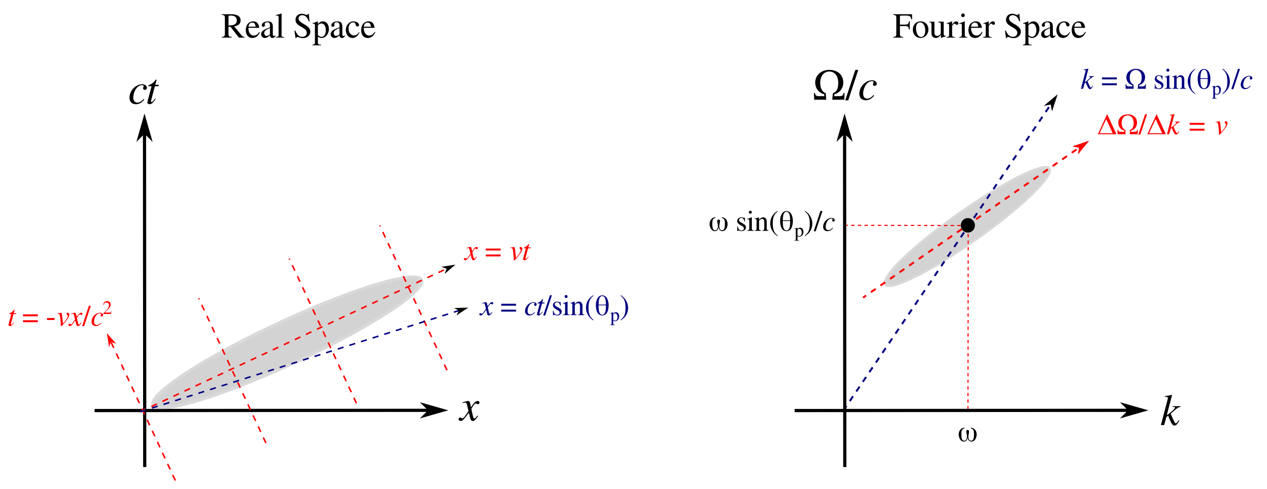

Supplementary Figure 7: **Single patch diffraction:** The behaviour of the diffraction pattern for a one–pump illumination is found through (left) assuming a space–time pattern of reflectivity inclined along the line $x = vt$, upon which is imprinted the phase of the probe field. The Fourier transform of this function is a patch in Fourier space, inclined relative to the angle of the probe $k = (\Omega/c)\sin(\theta_{\rm p})$.

Single extended patch

For the case of a single pump beam incident onto the surface of the multilayer, a single patch of the surface becomes reflecting along the space–time line $x = vt$, indicated as a shaded region Supplementary Figure 7. The incidence angle of the pump, $\theta_{1,2}$, determines the angle of this reflecting patch via $v = c/\sin(\theta_{1,2})$. We thus take the field on the surface of the ITO to have the form

$$H_y(x, z = 0, t) = r\left(x - \frac{v}{c}\tau, \tau + \frac{v}{c}x\right) {\rm e}^{{\rm i}\frac{\omega}{c}(\sin(\theta_p)x - \tau)} \tag{37}$$

where r is the complex reflection amplitude, $\theta_{\rm p}$ and ω are the central angle and frequency of the probe respectively and $\tau = ct$. We assume the patch of reflectivity is extended parallel to the line $x' = x - vt = 0$, where the first argument of r is constant, whereas it is relatively confined along the orthogonal lines parallel to $\tau' = \tau - vx/c = 0$, where the second argument is constant. Taking the Fourier transform of the surface field (37) we

find,

$$\tilde{H}_y(k,z=0,\Omega) = \int_{-\infty}^{\infty}\mathrm{d}x\int_{-\infty}^{\infty}\mathrm{d}\tau\, r\left(x-\frac{v}{c}\tau,\tau+\frac{v}{c}x\right)\mathrm{e}^{\mathrm{i}\frac{\omega}{c}(\sin(\theta_p)x-\tau)}\mathrm{e}^{-\mathrm{i}\left(kx-\frac{\Omega}{c}\tau\right)}$$

$$= \frac{1}{1+\frac{v^2}{c^2}}\int_{-\infty}^{\infty}\mathrm{d}x'\int_{-\infty}^{\infty}\mathrm{d}\tau'\, r\left(x',\tau'\right)\mathrm{e}^{-\mathrm{i}\left(\Delta k\frac{(x'+\frac{v}{c}\tau')}{1+v^2/c^2}-\frac{\Delta\Omega}{c}\frac{\tau'-\frac{v}{c}x'}{1+v^2/c^2}\right)} \tag{38}$$

$$= \frac{1}{1+\frac{v^2}{c^2}}\tilde{r}\left(\frac{\Delta k+\frac{v}{c^2}\Delta\Omega}{1+\frac{v^2}{c^2}},\frac{\frac{\Delta\Omega}{c}-\frac{v}{c}\Delta k}{1+\frac{v^2}{c^2}}\right) \tag{39}$$

where $\Delta k = k-\frac{\omega}{c}\sin(\theta_p)$ and $\Delta\Omega = \Omega-\omega$. Given the strong/weak dependence of r on it's first/second argument, in Fourier space the transformed amplitude $\tilde{H}_y$ is extended along the line parallel to $\Delta\Omega = v\Delta k$, and is narrow along the axis $\Delta k = -\frac{v}{c^2}\Delta\Omega$. In terms of diffraction angles $k = \frac{\omega}{c}\sin(\theta)$ the line along which the Fourier amplitude is extended is given by,

$$\Delta\Omega = v\Delta k$$
$$\rightarrow \Omega = \omega\left(\frac{1-\frac{v}{c}\sin(\theta_p)}{1-\frac{v}{c}\sin(\theta)}\right). \tag{40}$$

For small angles away from the probe $\theta = \theta_p+\delta\theta$, $\delta\theta \ll 1$ we can expand the sine in the above formula to give,

$$\frac{\Omega}{\omega} = 1+\frac{\frac{v}{c}\cos(\theta_p)}{1-\frac{v}{c}\sin(\theta_p)}\delta\theta$$
$$\rightarrow \frac{\Delta\Omega}{\omega} = \frac{\frac{v_r}{c}\cos(\theta_p)}{1-\frac{v_r}{v_p}}\delta\theta \tag{41}$$
$$\rightarrow \frac{\Delta\Omega}{\omega} \propto \frac{\delta\theta}{1-\frac{v_r}{v_p}}, \tag{42}$$

showing that the incident spectrum will be spread over a line with gradient $(v/c)\cos(\theta_p)/(1-(v/c)\sin(\theta_p))$. This gradient is positive (negative) when the modulation is moving faster (slower) than the probe, as indicated in the final proportionality (42). Eq. (41) is compared to both experiment and the full operator theory in the main text.

Two patches

A very similar argument to that of the single extended patch can be given when both pump beams are simultaneously incident on the surface. As in Eq. (37), we write the field

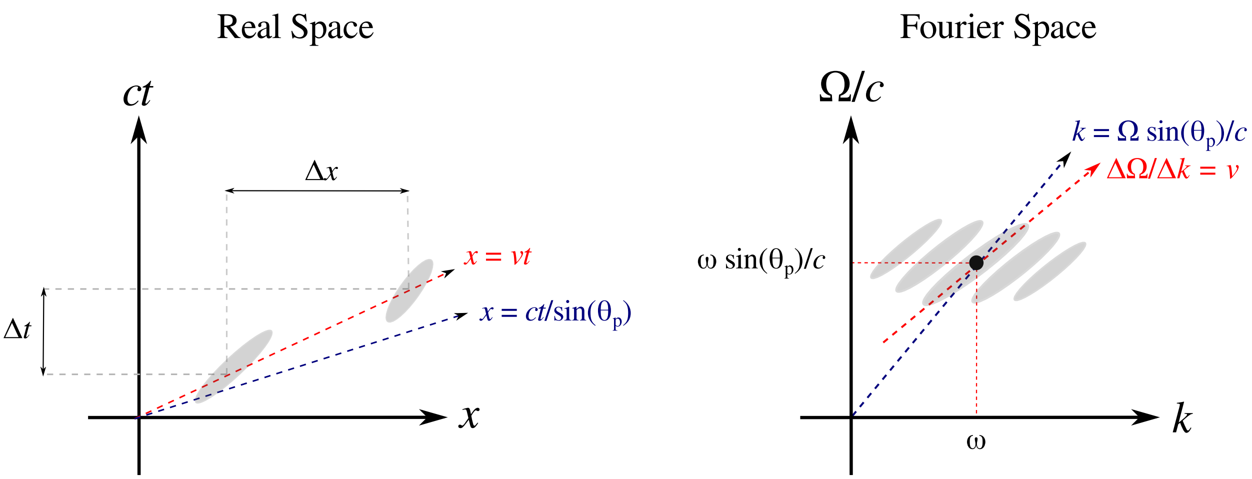

Supplementary Figure 8: **Two patch diffraction:** As described in Supplementary Figure 7, we model the diffraction as two isolated patches of reflectivity in space–time, separated by a distance Δx and time Δt. In Fourier space this consists of a series of interference fringes, tilted with $\Delta\Omega/\Delta k = v = \Delta x/\Delta t$.

on the surface as

$$H_y(x, z = 0, t) = [r_1(x, ct) + r_2(x - \Delta x, ct - c\Delta t)]\mathrm{e}^{\mathrm{i}\frac{\omega}{c}(\sin(\theta)x - ct)}, \tag{43}$$

with r_1 and r_2 describing the reflection amplitudes of the two slits, as shown in Supplementary Figure 8. Both functions r_1 and r_2 are centred around the origin of their space and time arguments (i.e. in Eq. (43, patch 1 is centred around $x = t = 0$, and patch 2 around $x = \Delta x$ and $t = \Delta t$). Performing the Fourier transform of the surface field (43) we have

$$\begin{aligned}\tilde{H}_y(k, z = 0, \Omega) &= \int_{-\infty}^{\infty} \mathrm{d}x \int_{-\infty}^{\infty} \mathrm{d}\tau\, [r_1(x, \tau) + r_2(x - \Delta x, \tau - c\Delta t)]\mathrm{e}^{\mathrm{i}\frac{\omega}{c}(\sin(\theta)x - \tau)}\mathrm{e}^{-\mathrm{i}\left(kx - \frac{\Omega}{c}\tau\right)} \\ &= \tilde{r}_1(\Delta k, \Delta\Omega/c) + \mathrm{e}^{-\mathrm{i}(\Delta k\Delta x - \Delta\Omega\Delta t)}\,\tilde{r}_2(\Delta k, \Delta\Omega/c),\end{aligned} \tag{44}$$

where Δk and $\Delta\Omega$ are defined as in the previous section. Taking the absolute value squared of (44), we obtain the Fourier representation of the intensity

$$\begin{aligned}\left|\tilde{H}_y(k, z = 0, \Omega)\right|^2 &= |\tilde{r}_1(\Delta k, \Delta\Omega/c)|^2 + |\tilde{r}_2(\Delta k, \Delta\Omega/c)|^2 \\ &+ 2|\tilde{r}_1(\Delta k, \Delta\Omega/c)||\tilde{r}_2(\Delta k, \Delta\Omega/c)| \cos\left(\Delta k\Delta x - \Delta\Omega\Delta t + \phi_{12}(\Delta k, \Delta\Omega/c)\right)\end{aligned} \tag{45}$$

where $\phi_{12} = \arg[\tilde{r}_1/\tilde{r}_2]$ is the phase difference between the reflection amplitudes of the patches. Equation (45) shows that the diffraction pattern from the surface consists of an

overall envelope $|\tilde{r}_1|^2 + |\tilde{r}_2|^2$ within which there is a set of interference fringes in wave–vector and frequency. The central maximum of this interference pattern occurs when

$$\Delta k \Delta x - \Delta\Omega \Delta t = -\phi_{12} \tag{46}$$

Assuming negligible phase difference between the patches, $\phi_{12} \sim 0$, and re–writing the above equation in terms of angles we predict the maximum to lie along the line,

$$\frac{\Omega}{\omega} = 1 + \frac{\frac{v}{c}\cos(\theta_p)}{1 - \frac{v}{c}\sin(\theta_p)}. \tag{47}$$

where $v = \Delta x/\Delta t$, and we have expanded around the probe angle ($\theta = \theta_p + \delta\theta$). Note that this is the same slope as predicted for the extended reflecting patch (41) but with the velocity now given by the ratio of the spatial and temporal displacement of the patches.

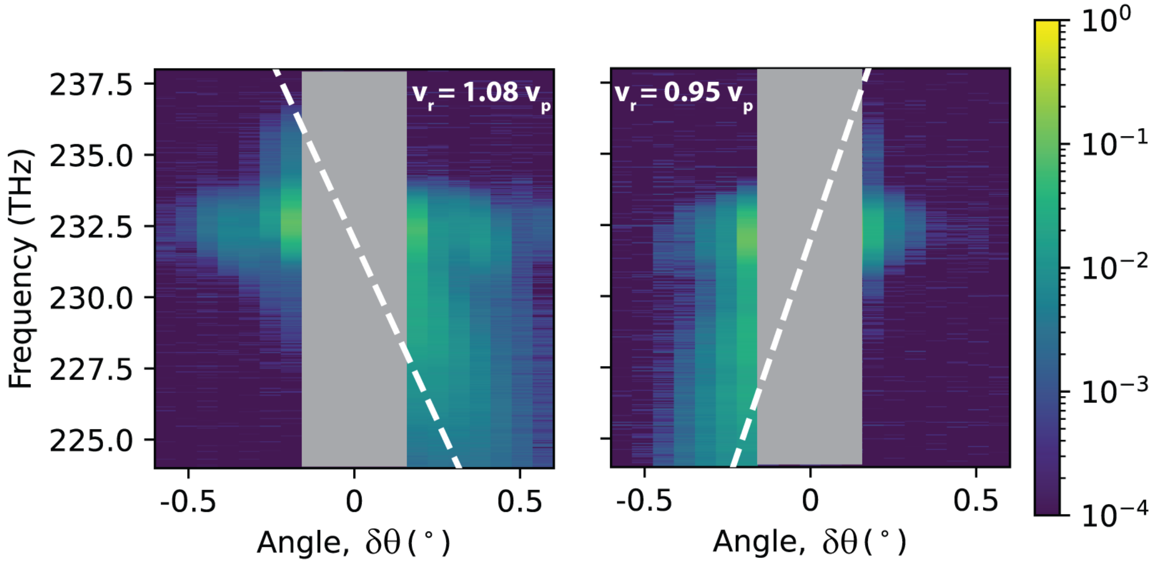

Supplementary Figure 9: Detecting hyperspectra in the wide field. Hyperspectra for space-time diffraction under the same conditions as the single extended modulations studied in the main text but omitting the reflected signal to increase the dynamic range. The z axis of these plots is normalised to the respective scans in the main text.

SUPPLEMENTARY DISCUSSION

The Dynamic Range of Measurements

The dynamic range, the ratio of the largest to smallest measurable signal, for the measured hyperspectra of the space-time diffracted probe is approximately 20 dB, a property of the spectrometer and experimental conditions. By consequence of this limiting dynamic range, the weaker diffracted signal is muted by the much larger reflected intensities, obscuring details and the extent of the diffraction signature. However, by limiting the angular range of a scan to the regions away from the reflected probe beam the details of the diffracted light are more clear, this was a tactic used when investigating the diffraction from a pair of modulation. Moreover, it can also be used to further investigate the diffraction from a single modulation as displayed in Supplementary Figure 9, using the same conditions as the scans studied in the main text. In these plots the shift in the diffraction pattern's angular centre of mass is clearly evident.

Reconfigurable Space-Time Diffraction

Discrete synthetic motion represents a platform for generating space-time diffraction that may be readily reconfigured and programmed. Here we demonstrate that the momentum-frequency fringes that result from space-time double slit diffraction may be generated with tuneable gradient and period. The gradient of the diffraction fringes can be controlled by tuning v_r, as depicted in Supplementary Figure 10A. Notably, when $v_r = v_p$ as in configuration ”2”, we observe a separable space-time transformation that gives rise to vertical fringes in momentum-frequency space. These oscillations in momentum are signatures of diffraction in space alone. On the other hand, we exhibit control over the period of the generated momentum-frequency fringes by controlling the separation between the two modulations in space-time, see Supplementary Figure 10B. Akin to configuration 2, here we also demonstrate a transformation that is very close to the separable double-slit diffraction in time only *(5)*, which offers horizontal fringes.

Scattering from a moving particle

We now compare the theory with light scattering from a real particle. Consider a small particle moving with velocity $v\hat{x}$ illuminated by a plane wave with magnetic field

$$\mathbf{H}(\mathbf{r}, t) = H_0 \hat{y} \exp\left[i(k_x x + k_z z - k_0 ct)\right] . \tag{48}$$

where the frequency is $\omega_0 = k_0 c$ and $k_x^2 + k_z^2 = k_0^2$. In the frame of the moving particle, the magnetic field becomes

$$\mathbf{H}'(\mathbf{r}', t') = H_0' \hat{y}' \exp\left\{i\left[\gamma(k_x - \beta k_0)x' + k_z z' - \gamma(k_0 - \beta k_x)ct'\right]\right\} . \tag{49}$$

where $\beta = v/c, \gamma = (1-\beta^2)^{-1/2}$. The frequency in the moving frame is thus

$$\omega' = \gamma(k_0 - \beta k_x)c\,. \tag{50}$$

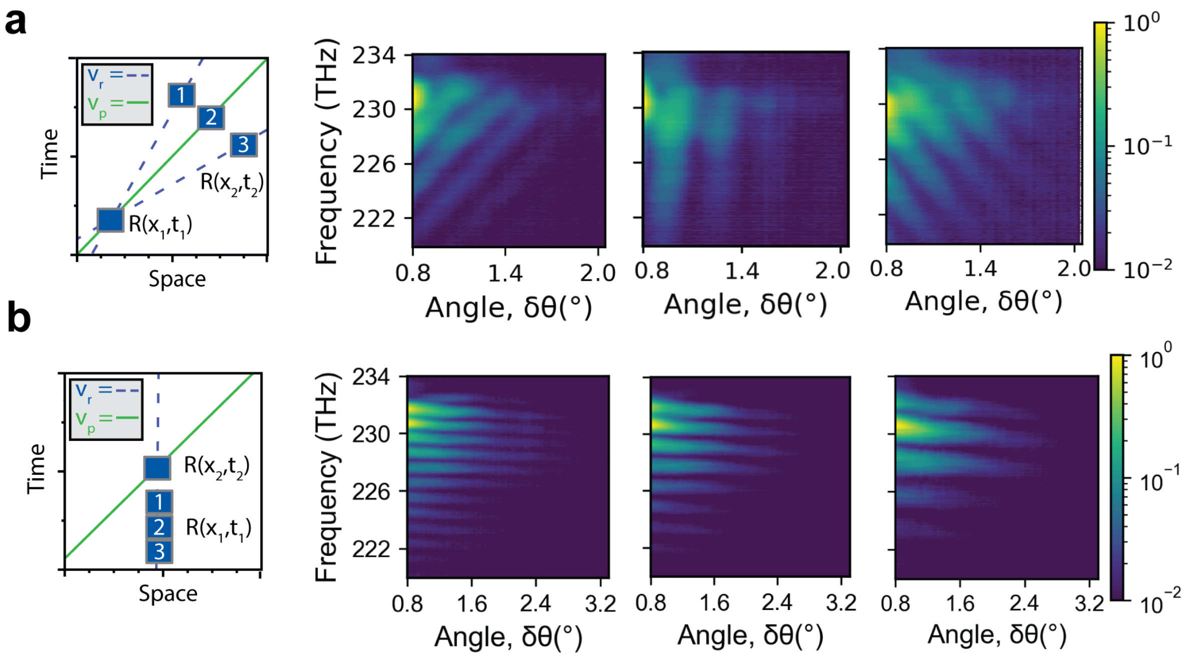

Supplementary Figure 10: Reconfigurable space-time double slit diffraction. (**a**) By tuning the synthetic velocity (see sketch, left) we demonstrate control over the gradient of the diffraction fringes. (**b**) Conversely, the period of the diffraction fringes may be controlled by tuning the separation of the two modulations in space-time (see sketch, left).

The particle now scatters light at the same frequency $\omega' = ck'$. Assuming Rayleigh scattering, using the angular spectral decomposition of the dipole field, the scattered field in the moving frame in the direction of reflection can be expressed as the superposition of plane waves of the form

$$\mathbf{H}'_{\mathbf{sc}}(\mathbf{r}, t) = \hat{y}' \int dk'_x A(k'_x) \exp\left[i(k'_x x' - k'_z z' - k' ct')\right] , \tag{51}$$

where $k_x'^2 + k_z'^2 = k'^2$ and we limit ourselves to waves with $k'_y = 0$. Transforming the scattered fields back to the rest frame,

$$\mathbf{H}_{\mathbf{sc}}(\mathbf{r}, t) = \hat{y} \int dk'_x A(k_x) \exp\left\{i\left[\gamma(k'_x + \beta k')x - k'_z z' - \gamma(k' + \beta k'_x)ct\right]\right\} . \tag{52}$$

We can now use this expression to relate the scattering angle and frequency in the rest frame,

$$\omega_{sc}(k'_x) = \gamma(k' + \beta k'_x) \tag{53}$$

$$\sin[\theta_{sc}(k'_x)] = \frac{\beta + k'_x/k'}{1 + \beta k'_x/k'} = \beta + \frac{1 - \beta^2}{\beta + k'/k'_x} . \tag{54}$$

For $\beta < 1$, both the detection angle and frequency are monotonically increasing with the in-plane component of the wavevector k'_x in the moving frame. Therefore we always see an increasing frequency shift with angle in the rest frame (Supplementary Figure 11, blue curve). The theory for scattering from the real particle (solid lines) matches Eq. (41) based on Fourier theory (dashed lines) exactly.

Faster-than-light particles

Although the above analysis is only valid for $v < c$, we can extend the algebra for $v > c$. In this case, we have $\gamma = i(\beta^2 - 1)^{-1/2}$ and the frequency in the moving frame,

$$\omega' = \gamma |k_0 - \beta k_x| c . \tag{55}$$

is now imaginary. So is $k' = \omega'/c$. To obtain propagating waves in the rest frame (scattered k_x, k_z real), we note from Eq. 52 that k'_x needs to be imaginary and k'_z real. Setting $k_x = i\kappa_x$, we need to satisfy the dispersion relation

$$(i\kappa_x)^2 + k_z^2 = k'^2 \implies \kappa_x^2 = |k'|^2 + k_z^2 . \tag{56}$$

The relation between scattering angle and frequency in the rest frame is given by

$$\omega_{sc}(\kappa_x') = |\gamma(k' + i\beta\kappa_x')| \ , \tag{57}$$

$$\sin[\theta_{sc}(\kappa_x')] = \pm\gamma\frac{i\kappa_x' + \beta k'}{\omega_{sc}/c} \ , \tag{58}$$

where the the modulus is taken to keep the frequency positive, sign is chosen to match the sign of the quantity inside the modulus in Eq. 57.

The frequency increases or decreases with the angle now depending on whether the particle speed is lower (Supplementary Figure 11, orange curve) or higher (green curve) than the probe speed $v_p = \omega_0/k_x$. Once again, the theory for real particles (solid lines) matches Fourier theory (dashed lines) exactly.

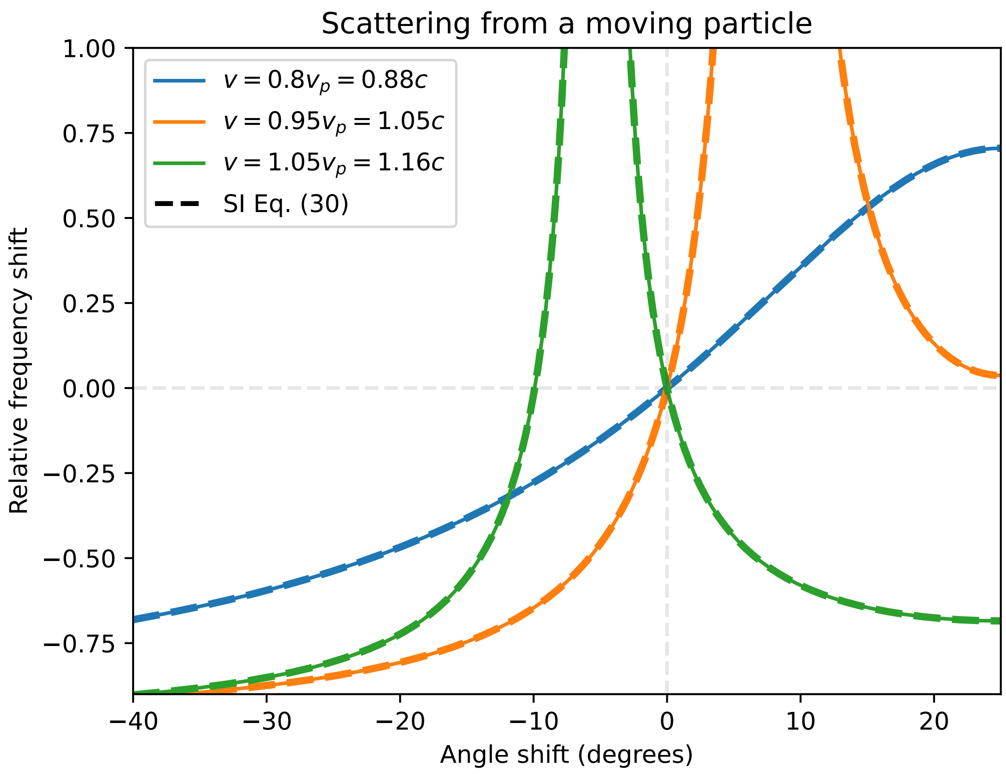

Supplementary Figure 11: Angular dispersion under scattering from a moving particle with velocity equal to that of the continuous modulations induced by pump 1 (green) and 2 (orange), alongside a particle with sub-luminal motion. All demonstrate perfect agreement with the Fourier theory.

Agreement with Fourier analysis

We can understand the match between the above Doppler shift and the preceding Fourier analysis of the modulated reflectivity as follows: in the rest frame of the moving reflective patch the incident frequency is given by Eq. (50), $\omega' = \gamma(\omega_0 - vk_x)$, which is scattered into a new wave with the same frequency, but modified wavevector component k'_x. Transforming this frequency and wavevector back to the lab frame gives the frequency of the scattered wave,

$$\begin{aligned} \omega_{\rm sc} &= \gamma(\omega' + vk'_x) \\ k_{\rm sc} &= \gamma\left(k'_x + \frac{v\omega'}{c^2}\right) \\ \rightarrow \omega_{\rm sc} &= \gamma\left(\gamma(1 - v^2/c^2)(\omega_0 - vk_x) + v\left(\frac{k_{\rm sc}}{\gamma}\right)\right) \\ \rightarrow \omega_{\rm sc} - \omega_0 &= v(k_{\rm sc} - k_x) \end{aligned} \tag{59}$$

the final line of which is the same expression derived earlier in Eq. (40). Note that the factors of γ cancel out from this result, meaning that the predicted frequency shift doesn't rely on any choice we make for γ when $v > c$.

Lorentz transform into a temporal frame

One way to avoid the imaginary quantities in the Lorentz transform is to work in a frame where the modulation has infinite speed and therefore behaves as a purely temporal modulation [7]. This involves moving to a frame with speed $c^2/v\hat{x}$, so that $\beta = c/v < 1$. We can follow the algebraic derivation in the previous section.

Under this transformation, the key difference is that the purely temporal modulation scatters a spectrum of frequencies, all with the same parallel component of the wavenumber,

$$k'_x = \gamma(k_x - \beta\omega_0/c)\,. \tag{60}$$

On transforming back to the rest frame, the scattered frequency and parallel component of the wavevector corresponding to the frequency ω' in the moving frame become,

$$\begin{aligned} \omega_{sc}(\omega') &= \gamma(\omega' + \beta k'_x c)\,, \\ k_{x,sc}(\omega') &= \gamma(k'_x + \beta\omega'/c)\,. \end{aligned} \tag{61}$$

With some algebraic manipulations,

$$\begin{aligned}
\omega_{sc}(\omega') - \omega_0 &= \gamma\omega' + \gamma\beta k'_x c - \omega_0 \\
&= \gamma\left[\frac{c}{\beta}\left(\frac{k_{x,sc}}{\gamma} - k'_x\right)\right] + \gamma\beta k'_x c - \omega_0 \\
&= \frac{c}{\beta}k_{x,sc} - \frac{c}{\beta\gamma}k'_x - \omega_0 \\
&= v k_{x,sc} - v\left(k_x - \beta\omega_0/c\right) - \omega_0 \\
\omega_{sc} - \omega_0 &= v(k_{x,sc} - k_x)\,. \qquad (62)
\end{aligned}$$

This matches the results from the Fourier theory as well as the Lorentz transformation into the moving frame, thus providing an alternative description for the space-time diffraction.

Diffraction from separable and non–separable reflectivity modulations

Separable space–time reflectivity modulations $r(x,t) = r(x) \times r(t)$ do not give us access to all possible frequency–momentum transformations of an incident electromagnetic wave, being equivalent to a product of convolution operations on the incident spectrum. An illustration is given in Supplementary Figure 12, where we show that a separable reflectivity modulation generates a set of horizontal and vertical interference fringes, whereas a non–separable modulation similar to an object in motion, generates interference fringes that are neither parallel to the ω or k axes.

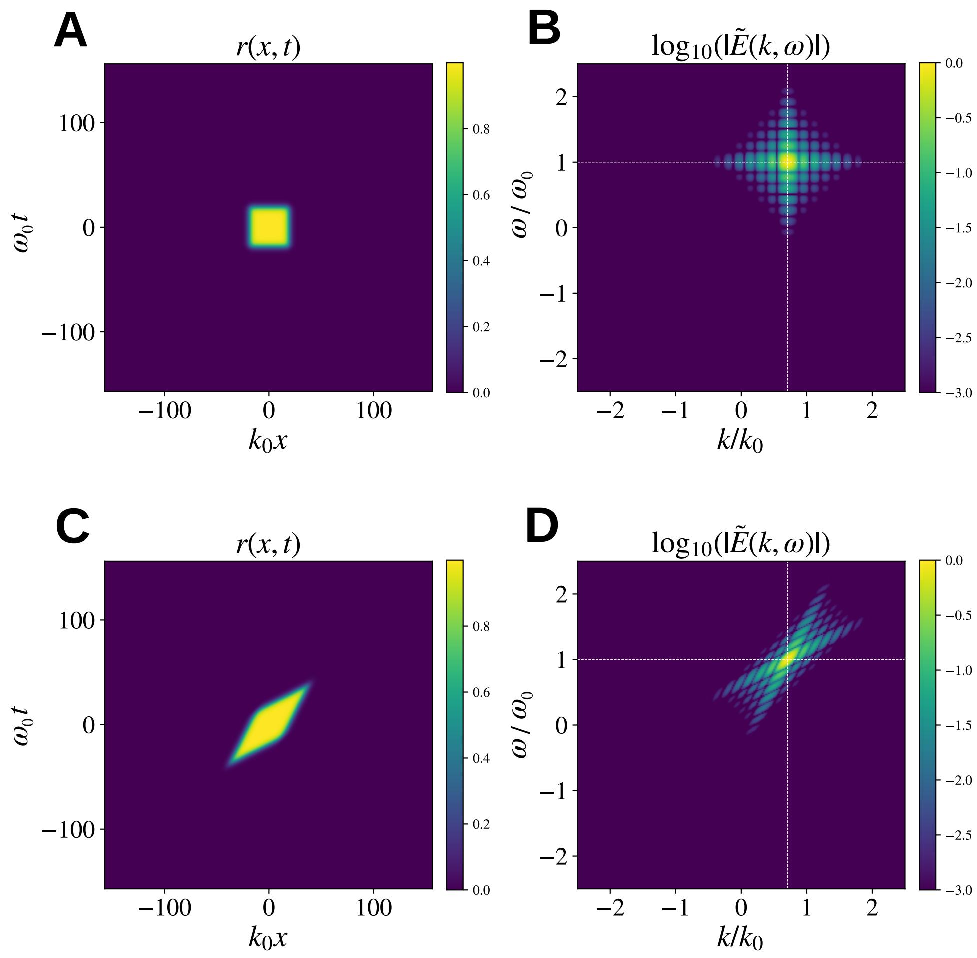

Supplementary Figure 12: (a–b) Separable reflectivity modulation $r(x,t) = r_x \times r_t$, with $r_x = \frac{1}{4}(1 + \tanh((x + L/2)/D_x))(1 + \tanh((L/2 - x)/D_x))$ and $r_t = \frac{1}{4}(1 + \tanh((t + T/2)/D_t))(1 + \tanh((T/2 - t)/D_t))$, with $k_0 L = 6\pi\lambda$, $k_0 D_x = \pi$, $\omega_0 T = 6\pi$, and $\omega_0 D_t = \pi$, where ω_0 and k_0 are the frequency and wave–number of the incident wave respectively. The angle of incidence is $\theta = 45°$ (incident frequency and in–plane wave–vector indicated as dashed lines). (c–d) Parameters as for the first two panels, but the reflectivity modulation is transformed as $x \to x - t/2$ and $t \to t - x/2$, which is now non–separable.

SUPPLEMENTARY REFERENCES

[1] Tirole, R. *et al.* Saturable time-varying mirror based on an epsilon-near-zero material. *Physical Review Applied* **18**, 054067 (2022).

[2] Un, I.-W., Sarkar, S. & Sivan, Y. Electronic-based model of the optical nonlinearity of low-electron-density drude materials. *Phys. Rev. Appl.* **19**, 044043 (2023).

[3] Secondo, R., Khurgin, J. & Kinsey, N. Absorptive loss and band non-parabolicity as a physical origin of large nonlinearity in epsilon-near-zero materials. *Opt. Mat. Exp.* **10**, 1545 (2020).

[4] Blair, S. F. J., Male, J. S., Cavill, S. A., Reardon, C. P. & Krauss, T. F. Photonic characterisation of indium tin oxide as a function of deposition conditions. *Nanomaterials* **13** (2023).

[5] Ko, D. Y. K. & Sambles, J. R. Scattering matrix method for propagation of radiation in stratified media: attenuated total reflection studies of liquid crystals. *JOSA A* **5**, 1863–1866 (1988).

[6] Horsley, S. A. R., Galiffi, E. & Wang, Y.-T. Eigenpulses of dispersive time-varying media. *Physical Review Letters* **130**, 203803 (2023).

[7] Deck-Léger, Z.-L., Chamanara, N., Skorobogatiy, M., Silveirinha, M. G. & Caloz, C. Uniform-velocity spacetime crystals. *Advanced Photonics* **1**, 056002 (2019). Publisher: SPIE.
